# Dietary intake of eicosapentaenoic and docosahexaenoic acids is linked to gray matter volume and cognitive function in elderly

**DOI:** 10.1007/s11357-012-9453-3

**Published:** 2012-07-13

**Authors:** Olga E. Titova, Per Sjögren, Samantha J. Brooks, Joel Kullberg, Erika Ax, Lena Kilander, Ulf Riserus, Tommy Cederholm, Elna-Marie Larsson, Lars Johansson, Håkan Ahlström, Lars Lind, Helgi B. Schiöth, Christian Benedict

**Affiliations:** 1 Department of Neuroscience, Uppsala University, Uppsala, Sweden; 2 Department of Public Health and Caring Sciences, Section of Clinical Nutrition and Metabolism, Uppsala University, Uppsala, Sweden; 3 Department of Radiology, Uppsala University, Uppsala, Sweden; 4 Department of Public Health and Caring Sciences/Geriatrics, Uppsala University, Uppsala, Sweden; 5 Department of Medical Sciences, Uppsala University, Uppsala, Sweden

**Keywords:** Omega-3 polyunsaturated fatty acids, Elderly, Cognitive function, Magnetic resonance imaging

## Abstract

In the present study, we tested whether elderly with a high dietary intake of eicosapentaenoic acid (EPA) and docosahexaenoic acid (DHA) would have higher cognitive test scores and greater brain volume than those with low dietary intake of these fatty acids. Data were obtained from the Prospective Investigation of the Vasculature in Uppsala Seniors (PIVUS) cohort. The dietary intake of EPA and DHA was determined by a 7-day food protocol in 252 cognitively healthy elderly (122 females) at the age of 70 years. At age 75, participants' global cognitive function was examined, and their brain volumes were measured by magnetic resonance imaging (MRI). Three different multivariate linear regression models were applied to test our hypothesis: model A (adjusted for gender and age), model B (additionally controlled for lifestyle factors, e.g., education), and model C (further controlled for cardiometabolic factors, e.g., systolic blood pressure). We found that the self-reported 7-day dietary intake of EPA and DHA at the age of 70 years was positively associated with global gray matter volume (*P* < 0.05, except for model C) and increased global cognitive performance score (*P* < 0.05). However, no significant associations were observed between the dietary intake of EPA and DHA and global white matter, total brain volume, and regional gray matter, respectively. Further, no effects were observed when examining cognitively impaired (*n* = 27) elderly as separate analyses. These cross-sectional findings suggest that dietary intake of EPA and DHA may be linked to improved cognitive health in late life but must be confirmed in patient studies.

## Introduction

Eicosapentaenoic acid (EPA, 20:5n-3) and docosahexaenoic acid (DHA, 22:6n-3), also known as omega-3 fatty acids, are polyunsaturated fatty acids that are mainly found in fish and food of marine origin. In humans, these fatty acids can be produced by enzymatic elongation and desaturation of their precursor α-linolenic acid (ALA, C18:3n-3). However, this conversion is very inefficient (Burdge 2004). Bearing in mind that EPA and DHA form major structural lipid components of neural membranes in the central nervous system (CNS) (Kawakita et al. 2006), a low intake of these omega-3 polyunsaturated fatty acids may contribute to cognitive aging in elderly. Supporting this view, epidemiological data have demonstrated that reduced dietary intake of EPA and DHA is associated with accelerated cognitive decline or the development of dementia, including Alzheimer's disease (Kalmijn et al. 1997, 2004), (van Gelder et al. 2007), (Samieri et al. 2008), (Huang 2010), (Dangour et al. 2010), (Cederholm and Palmblad 2010). These findings support the view that a high dietary intake of EPA and DHA during late adulthood could serve as a promising public health intervention to counteract cognitive decline facing aging modern societies. However, there are also negative findings demonstrating that the dietary intake of omega-3 fatty acids does not affect the rate of cognitive decline and the risk to develop dementia among elderly (Engelhart et al. 2002), (Devore et al. 2009). For instance, in the Rotterdam Study which initially included 5,395 participants (age, ≥55 years) who were free of dementia, a follow-up investigation ten years later revealed that the dietary intake of omega-3 fatty acids did not appear to be associated with long-term dementia risk (Devore et al. 2009). A potential drawback of many studies that also warrants attention is the inclusion of participants with heterogeneous age.

Against this background, the present community-based epidemiological study with a large cohort addressed the hypothesis that a high dietary intake of EPA and DHA in men and women at the age of 70 years was associated with both greater brain volume and better cognitive performance. To this aim, the daily intake of EPA and DHA was determined by means of a 7-day food protocol at the age of 70 years. To add further support to self-report measures of food intake, the plasma fatty acid composition was also determined. Factors that are known to affect cognitive performance and brain structure in the general population (education, self-reported physical activity, serum concentration of low-density cholesterol, body mass index, systolic blood pressure, and insulin resistance were measured at the age of 70 years so that they could be included as covariates. Five years after the dietary records were obtained, all participants underwent a magnetic resonance imaging (MRI) brain scan. Further, as a test of general cognitive function, the 7-min screen (7MS) test was administered (Solomon et al. 1998).

## Subjects and methods

### Design

**Fig. 1:**
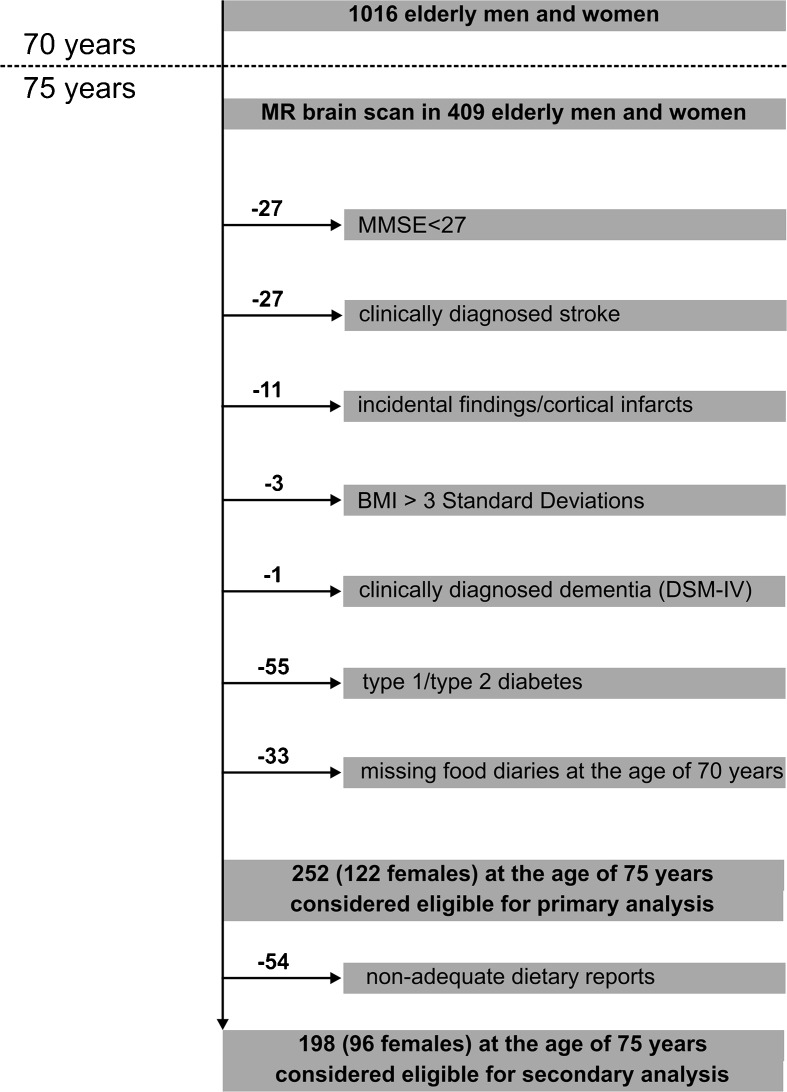
Subject inclusionary criteria and sample sizes

The study is based on the prospective cohort study “Prospective Investigation of the Vasculature in Uppsala Seniors” (PIVUS) (Lind et al. 2005), that initially included 1,016 (50 % females) individuals aged 70 living in the community of Uppsala, Sweden. The primary study aim was to investigate endothelial function and arterial compliance in a random sample of elderly subjects. After their inclusion between 2001 and 2003 during which a 7-day food diary was obtained, 827 subjects from the initial cohort agreed to participate in a follow-up investigation 5 years later (81.4 % response rate), i.e., when they were 75 years old. Of the individuals who were re-investigated at the age of 75 years, a subsample of 409 elderly agreed to participate in a magnetic resonance imaging (MRI) scan of their brains (49.5 % of the cohort that was re-investigated at the age of 75 years). Of this number, 252 elderly satisfied all criteria for this study, including cognitively normal clinical status (identified by an MMSE score greater than 26) (Folstein et al. 1975), absence of strokes or neurologic diseases (e.g., tumors) at ages 70 and 75, valid measures from the MRI brain scan, and an availability of the food diary record at the age of 70 years (Flow-chart, please see Fig. 1). Exclusions were administered to minimize the confounding effects of variables related to our main question: does the dietary intake of DHA and EPA at the age of 70 years relate to cognition and brain structure 5 years later among cognitively healthy elderly. In addition, a subpopulation (*n* = 198) was created in which individuals with unreliable dietary records were excluded (see below).

The study was approved by the Ethics Committee of the University of Uppsala and the participants gave informed consent to participate.

### Dietary assessment

Dietary habits were determined as previously published (Sjogren et al. 2010). Briefly, the participants were given oral instructions by a dietitian on how to perform the dietary registration over seven consecutive days, and the amounts consumed were reported in household measurements or specified as portion sizes. The daily intake of EPA and DHA was calculated by using a database from the Swedish National Food Agency containing about 1,500 food items, drinks, and recipes. Non-adequate reporters of energy intake were identified using the Goldberg cutoff (Black 2000) taking the level of physical activity and basal metabolic rate into consideration.

### Assessments of physical activity and education at the age of 70 years

Physical activity (PA) was divided into light and hard exercise and classified as number of activities with a duration of at least 30 min per week. The participants were asked how many times per week they performed light (e.g., walking, gardening), respectively hard exercise (e.g. running, swimming) for at least 30 min. Based on the responses to these questions, four PA categories were constructed: very low, low, medium, and high.

The educational level (i.e., primary school, secondary school, and university level) for each subject was assessed by means of a standardized questionnaire.

### Clinical and biochemical investigation at the age of 70 years

Blood samples were collected in the morning after an overnight fast using Architect (Abbott, Abbot Park, IL, USA) for biochemical analysis, unless otherwise stated. Low-density lipoprotein (LDL) cholesterol was calculated using Friedewald's formula. Plasma glucose and serum insulin concentration values were used to calculate the homeostasis model assessment-insulin resistance (HOMA-IR; [(fasting plasma glucose × fasting serum insulin)/22.5]), a measure of insulin sensitivity (Wallace and Matthews 2002). The proportion of DHA and EPA in serum phospholipids was measured as previously described (Warensjo et al. 2009). Briefly, the samples were extracted in chloroform, separated by thin-layer -chromatography, trans-methylated and then separated by gas liquid chromatography with a system from Hewlett-Packard (Avondale, PA)) on a capillary column (Quadrex, New Heaven, CT, USA). Methyl ester standards (GLC- 68A, Nu Check Prep, Elysian, MN, USA) were used to identify the DHA and EPA. After recording height and weight allowing the calculation of the body mass index (BMI, kilogram per square meter), the subjects were supine in a quiet room maintained at a constant temperature, and the systolic blood pressure (BP) was measured by a calibrated mercury sphygmomanometer in the non-cannulated arm to the nearest mmHg.

### Cognitive measures

At the age of 75 years, a Swedish translation of the 7 min screen (7MS) test was administered to the participants by trained study nurses. This test is clinically used to screen for dementia and cognitive decline, and has been described in (Solomon et al. 1998). The 7MS consists of four brief cognitive tests:

- Benton temporal orientation—BTO (Benton 1983): In this test, the orientation for time is measured and quantified in terms of degree of error. The maximum score is 113 (10 error points for one year, 5 points for one month, 1 for the date and the day of a week, and 1 for each 30 min deviation in time).
- Enhanced cued recall—ECR (Grober et al. 1988): This declarative memory test requires the subject to recall 16 figures. The score is the total number of items recalled in both free and cued recall (range, 0–16).
- Clock drawing—CD (Freedman et al. 1994): This test is to examine the visuoconstruction. In detail, the subject has to draw the face of a clock and place the hands of the clock at a given time. The maximum score is 7 points.
- Categorical verbal fluency—VF: In this semantic test, the subject has to name as many different animals as possible in 1 min time.

The raw scores of the four subtests of the 7MS were summed with the logistic regression formula described previously (Solomon et al. 1998): ${\text{Ln}}\left[ {P/\left( {1 - P} \right)} \right] = 35.59-1.303*{\text{ECR}} - 1.378*{\text{VF}} + 3.298*{\text{BTO}} - 0.838*{\text{CD}}$, where *P* is the probability of having dementia. Solomon estimated the formula by using the scores of the four tests from the screening battery as predictor variables. The natural logarithm (ln) of *P*/(1 − *P*) is equal to the total 7MS score of the above logistic regression formula. The more negative the total raw score on the 7-min screening (7MS) test, the lower the probability of having dementia (3). For purposes of presentation, scores were inverted, such that the higher the score the better the performance on the 7MS.

### MRI acquisition and processing

At the age of 75 years, regional measures of brain volume were acquired with MRI. A high resolution 3D T1-weighted volumetric “Turbo Field Echo” (TFE) scan was acquired using a Philips 1.5 Tesla scanner (Gyroscan NT, Philips Medical Systems, Best, The Netherlands). The 3D gradient echo sequence was acquired with the scan parameters TR 8.6 ms, TE 4.0 ms, flip angle = 8°. Sagittal slices with a field of view of 240 mm, a slice thickness of 1.2 mm, and an in-slice resolution of 0.94 mm^2^ were reconstructed.

Images were processed using Voxel Based Morphometry (VBM), a technique that used statistical parametric mapping (SPM) to determine local concentrations of gray matter volumes on a voxel-by-voxel basis (Ashburner and Friston 2000). Gray matter was calculated by segmenting it from white matter and cerebrospinal fluid using the unified segmentation approach (Ashburner and Friston 2005). Following this segmentation procedure, probability maps of gray matter were “modulated” to account for the effect of spatial normalization, by multiplying the probability value of each voxel by its relative volume in native space before and after warping. Gray matter images based on probability maps at each voxel were normalized into Montreal Neurological Institute (MNI) standard space with a voxel size of 2 × 2 × 2 mm. Modulated images were smoothed with an 8-mm Full Width Half Maximum (FWHM) Gaussian kernel, in line with other recent VBM studies (Walther et al. 2010). This smoothing kernel was applied prior to statistical analysis, to reduce signal noise and to correct for image variability. VBM analyses were carried out using SPM8 (Functional Imaging Laboratory, University College London) (Ashburner and Friston 2000).

### Statistical analysis

For statistical evaluation, SPSS version 19.0 (SPSS Inc, Chicago, IL) was used. Data are presented as means ± SEMs and were analyzed using linear regression models. Normal distribution of all variables was confirmed by Kolmogorov–Smirnov (KS) testing. Since the 7-day food record approximates does not exactly correspond to the actual intake of EPA and DHA, the participants were divided into four groups according to their daily intake of EPA and DHA: very low (0.026–0.226 g; <25th percentile), low (0.228–0.387 g; 25th–50th percentile), medium (0.40–0.666 g; 50th–75th percentile), and high intake (0.667–1.910 g; >75th percentile). Gray matter, white matter, and total brain volumes are expressed as relative to the total intracranial volume (TIV). To test our hypothesis that the dietary intake of EPA and DHA is linked to greater brain volume and better cognitive performance, three linear regression models were administered, covarying for factors that have been previously described to be associated with our main measures in the general population:

Model A: adjusting for gender and exact age
Model B: model A + energy intake, education (Le Carret et al. 2003), and self-reported physical activity (Weuve et al. 2004)
Model C: model B + serum concentration of low-density cholesterol (Ward et al. 2010), BMI (Brooks et al. 2012), systolic blood pressure (Muller et al. 2010) and HOMA-IR (Benedict et al. 2012).

The three models were applied in our main study population as well as in the subpopulation of adequate reporters of energy intake.

In addition to global brain measures (i.e., gray matter, white matter, and total brain tissue), a voxel-based morphometry regression model was tested to examine as to whether the EPA and DHA intake is predictive for regional gray matter volumes, controlling for both gender and total intracranial volume (TIV, defined as the sum of gray matter, white matter, and cerebrospinal fluid). All clusters and peak voxels of gray matter T statistic brain maps reported were thresholded at a *P* value < 0.05 by using Family Wise Error (FWE). In order to avoid possible edge effects between different tissue types, we excluded from our analyses all voxels with gray matter probabilities of less than 0.1 (absolute threshold masking). The association between the DHA and EPA content in serum phopholipids and dietary intake of these fatty acids was specified by Spearman's rank test. Overall, a two-sided *P* value less than 0.05 was considered significant.

## Results

### Self-reported 7-day EPA and DHA intake is positively linked to gray matter volume and cognitive performance

Descriptive data of the study population are shown in Table 1. The basic linear regression model in the primary analysis revealed a positive association between the dietary intake of EPA and DHA at the age of 70 years and global gray matter volume five years later (Table 2). Subsequent regression model controlling for lifestyle factors (all measured at the age of 70 years) confirmed this significant association (Table 2). In the regression model C, further controlled for cardiometabolic confounders, the dietary intake of EPA and DHA was still positively linked to global gray matter volume. However, this association failed to reach the 5 % significance level (Table 2). In contrast, regression analysis did not produce any significant associations between the dietary intake of EPA and DHA on the one side and white matter volume and total matter volume on the other side (Table 2). Using whole-brain correction for multiple comparisons, voxel-based morphometry did not produce any regional differences in gray matter. Despite its association with global gray matter, the dietary intake of EPA and DHA was also positively linked to the 7MS score (i.e., the total score obtained on four cognitive subtests). This association remained significant in all models (Table 2).

**Table 1:** Descriptive statistics of participants, stratified according to their daily intake of eicosapentaenoic and docosahexaenoic acids

	EPA/DHA intake			
	Very low	Low	Medium	High
No. of subjects	63	63	63	63
No. of females	25	31	34	32
Educational level (*primary/secondary school/university; n/n/n*)	32/13/18	40/13/10	35/11/17	29/12/22
At 70 years				
Exact age	70.1 ± 0.0	70.2 ± 0.0	70.2 ± 0.0	70.1 ± 0.0
Intake of EPA/DHA (g/day)	0.13 ± 0.01	0.30 ± 0.01	0.52 ± 0.01	0.98 ± 0.03
Range of EPA/DHA intake (g/day)	0.026–0.226	0.228–0.387	0.400–0.666	0.667–1.910
Energy intake, KJ	8057 ± 333	7665 ± 236	8076 ± 254	8230 ± 271
BMI, in kg/m^2^	26.7 ± 0.5	26.0 ± 0.4	26.4 ± 0.5	26.7 ± 0.4
Blood glucose levels, in mmol/L (**A**)	4.95 ± 0.07	4.88 ± 0.05	4.83 ± 0.06	4.90 ± 0.06
Blood insulin levels, in μg/dL (**B**)	7.92 ± 0.52	8.58 ± 0.54	7.87 ± 0.78	7.65 ± 0.56
HOMA-IR (A*B/22.5)	1.77 ± 0.13	1.87 ± 0.12	1.57 ± 0.10	1.68 ± 0.13
Serum LDL cholesterol, in mmol/L	3.47 ± 0.13	3.41 ± 0.09	3.55 ± 0.09	3.55 ± 0.11
Systolic blood pressure, in mmHg	145 ± 3	144 ± 2	149 ± 3	146 ± 2
Self-reported Physical activity (four levels)	2.32 ± 0.10	2.29 ± 0.09	2.31 ± 0.10	2.28 ± 0.08
At 75 years				
Exact age	75.2 ± 0.0	75.3 ± 0.0	75.3 ± 0.0	75.3 ± 0.0
Gray matter volume, ml	574 ± 8	571 ± 9	565 ± 8	585 ± 7
White matter volume, ml	456 ± 6	438 ± 8	443 ± 8	447 ± 7
TIV, ml	1772 ± 16	1744 ± 19	1706 ± 20	1762 ± 19
MMSE (max score 30)	29.0 ± 0.1	28.8 ± 0.1	29.1 ± 0.1	29.1 ± 0.1
Benton temporal orientation	0.46 ± 0.19	0.54 ± 0.18	0.32 ± 0.07	0.38 ± 0.12
Enhanced cued recall (max score 16)	15.75 ± 0.09	15.68 ± 0.11	15.78 ± 0.07	15.87 ± 0.04
Clock drawing (max score 7)	6.4 ± 0.1	6.4 ± 0.1	6.5 ± 0.1	6.5 ± 0.1
Verbal fluency (words per min)	19.9 ± 0.7	20.1 ± 0.7	20.2 ± 0.6	21.9 ± 0.6

If not otherwise described, raw data are mean ± SEM. *Abbreviations*: *BMI*, body mass index; *DHA*, docosahexaenoic acid; *EPA*, eicosapentaenoic acid; *HOMA-IR*, homeostasis model assessment (Wallace and Matthews 2002); *LDL*, low-density lipoprotein cholesterol; *MMSE*, mini-mental state examination (Folstein et al. 1975); *TIV*, total intracranial volume (i.e., the sum of gray matter, white matter, and cerebrospinal fluid)

**Table 2:** The association between the daily intake of eicosapentaenoic and docosahexaenoic acid at the age of 70 years and cognitive function as well as brain volume 5 years later

	EPA/DHA very low *N* = 63	EPA/DHA Low *N* = 63	EPA/DHA Medium *N* = 63	EPA/DHA High *N* = 63	Model	β-value EPA/DHA	*P* value	Cohen's *ƒ* ^2^
Gray matter (% TIV), 75 years	32.4 ± 0.3	32.7 ± 0.4	33.1 ± 0.4	33.3 ± 0.4	Model A	0.14	**0.03**	0.06
					Model B	0.12	**<0.05**	0.07
					Model C	0.11	0.08	0.10
White matter (% TIV), 75 years	25.7 ± 0.3	25.1 ± 0.3	25.9 ± 0.3	25.3 ± 0.2	Model A	−0.01	0.93	0.02
					Model B	−0.02	0.74	0.05
					Model C	−0.03	0.63	0.08
Brain tissue (% TIV), 75 years	58.1 ± 0.5	57.8 ± 0.6	59.1 ± 0.5	58.6 ± 0.5	Model A	0.10	0.12	0.07
					Model B	0.08	0.20	0.09
					Model C	0.07	0.29	0.12
7MS (total score), 75 years	16.1 ± 1.1	16.1 ± 1.2	17.2 ± 0.9	19.5 ± 1.0	Model A	0.15	**0.02**	0.03
					Model B	0.14	**0.02**	0.16
					Model C	0.13	**0.03**	0.23

Raw data are mean ± SEM. To test our hypothesis that the dietary intake of eicosapentaenoic and docosahexaenoic acid (EPA and DHA, respectively) at the age of 70 years is linked to greater brain volume (measured by magnetic resonance imaging and analyzed by voxel-based morphometry, (Ashburner and Friston 2000)) and better cognitive performance (7-min screening, 7MS; (Solomon et al. 1998)) 5 years later. The following linear regression models were administered: *Model A*, adjusting for gender, age; *Model B*, controlling for gender, age, energy intake, education (Le Carret et al. 2003), and self-reported physical activity (Weuve et al. 2004); *Model C*, covarying for gender, age, energy intake, education, self-reported physical activity, serum concentration of low-density cholesterol (Ward et al. 2010), BMI (Brooks et al. 2012), systolic blood pressure (Muller et al. 2010) and HOMA-IR (Benedict et al. 2012). To control for individual differences in head size, the regression models testing brain volumes were additionally controlled for total intracranial volume (TIV, defined as the sum of gray matter, white matter, and cerebrospinal fluid). Note that brain tissue equals the sum of white matter and gray matter. Overall, a *p* value less than 0.05 was considered significant (significances are indicated in bold). The ƒ^2^ effect size measure was defined as: ƒ^2^ = *R* ^2^/(1 − *R* ^2^). By convention, ƒ^2^ effect sizes of 0.02, 0.15, and 0.35 are termed small, medium, and large, respectively (Cohen 1988)

Importantly, similar but slightly less significant functional and structural results were obtained when excluding 54 participants from the analyses who apparently did not report their dietary intake properly (Table 3).

**Table 3:** The association between the daily intake of eicosapentaenoic and docosahexaenoic acid at the age of 70 years and cognitive function as well as brain volume 5 years later (without those who apparently did not report their dietary intake properly)

	EPA/DHA very low *N* = 49	EPA/DHA Low *N* = 50	EPA/DHA Medium *N* = 50	EPA/DHA High *N* = 49	Model	β-value EPA/DHA (standardized)	*P* value	Cohen's *ƒ* ^2^
Gray matter (% TIV), 75 years	32.6 ± 0.4	32.7 ± 0.4	33.5 ± 0.4	33.5 ± 0.4	Model A	0.14	**<0.05**	0.08
					Model B	0.14	**0.04**	0.10
					Model C	0.14	0.06	0.11
White matter (% TIV), 75 years	25.7 ± 0.3	25.2 ± 0.4	26.2 ± 0.3	25.2 ± 0.2	Model A	−0.01	0.85	0.02
					Model B	−0.02	0.83	0.05
					Model C	−0.02	0.82	0.10
Brain tissue (% TIV), 75 years	58.3 ± 0.6	57.9 ± 0.6	59.7 ± 0.6	58.7 ± 0.5	Model A	0.09	0.18	0.08
					Model B	0.10	0.16	0.12
					Model C	0.09	0.20	0.14
7MS (total score), 75 years	16.5 ± 1.3	15.9 ± 1.3	18.3 ± 0.9	19.3 ± 1.2	Model A	0.14	**<0.05**	0.02
					Model B	0.12	0.07	0.16
					Model C	0.12	0.08	0.22

Raw data are mean ± SEM. Overall, a *p* value less than 0.05 was considered significant (significances are indicated in bold). For a detailed description, please see legend of Table 2

For all analyses presented, the interaction term of the dietary intake of EPA and DHA by gender did not reach significance. Regarding the cognitively impaired subgroup (*n* = 27), separate regression analyses revealed no significant association between the dietary intake of EPA and DHA on the one hand and cognitive functions and brain structure on the other hand (data are not shown).

### Correlation between DHA and EPA intake and their proportion in serum phospholipids

**Fig. 2:**
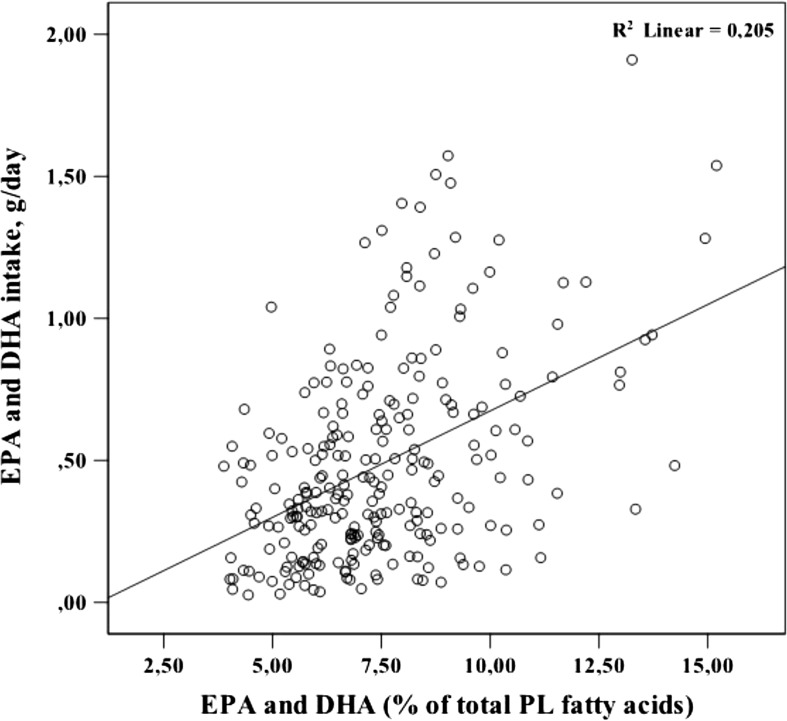
Positive correlation between daily intake of eicosapentaenoic and docosahexaenoic acids and their proportion in serum phospholipids. Correlational analyses between the estimated daily dietary intake of eicosapentaenoic acid (EPA) and docosahexaenoic acid (DHA) and the proportion of these fatty acids in circulating phospholipids (PL) at the age of 70 years. Spearman's rho = 0.40, *P* < 0.05; *N* = 252

Correlational analyses revealed that the estimated daily dietary intake of EPA and DHA was positively associated with the proportion of these fatty acids in circulating phospholipids at the age of 70 years (Spearman's *r* = 0.400, *p* < 0.0001; Fig. 2). The corresponding association in the subpopulation of only adequate reporters did also reach significance (Spearman's *r* = 0.362, *p* < 0.0001). There were no associations between plasma EPA or DHA with the performance on the 7MS or brain volume estimates.

## Discussion

Using data obtained from a large, community-based cohort of elderly Swedish men and women, we present a novel positive association between the dietary intake of EPA, DHA, brain function, and structure. Specifically, those whose diets were rich in EPA and DHA at the age of 70 years obtained significantly higher total scores on four standardized cognitive tests, and had significantly greater gray matter volumes 5 years later, than those whose diets were low in these fatty acids. Importantly, correlation analyses revealed that the estimated daily dietary intake of EPA and DHA was positively associated with the proportion of these fatty acids in circulating phospholipids, suggesting that the 7-days food record was a valid measure to approximate the habitual intake of EPA and DHA in our elderly sample. However, there was no significant relationship between plasma proportions of these fatty acids and cognitive tests or gray matter volumes, perhaps due to peripheral mechanisms that cause varied fluctuations in blood, and thus highlighting that some caution must be heeded when interpreting the results in brain. Further studies will be needed to establish and further elucidate whether consumption of omega-3 polyunsaturated fatty acids may have subtle but positive effects on both brain function and structure.

Findings from this study are in line with previous epidemiological studies. For instance, both high fish consumption and dietary intake of EPA and DHA have been shown to postpone cognitive decline in cognitively healthy elderly (van Gelder et al. 2007), (Kalmijn et al. 2004) (Albanese et al. 2009). In addition, previous interventional studies have revealed that DHA supplementation may improve learning and memory function in cognitively healthy elderly (Yurko-Mauro et al. 2010). Providing further evidence for a beneficial effect of omega-3 fatty acids on cognitive aging, a recent DHA intervention study over 6 months has indicated possible retardation of cognitive decline in patients with mild Alzheimer's disease (Freund-Levi et al. 2006). These studies, together with findings that both high plasma concentration of EPA and habitual high consumption of fish are associated with lower incidence of late-life dementia (Samieri et al. 2008), have received a recent increase in research focus. Nevertheless, the reader should bear in mind that other studies have not found beneficial effects of omega-3 fatty acids on mental health. For instance, in a randomized, double-blind, placebo-controlled trial including 302 individuals aged > or =65 years, EPA/DHA supplementation for 26 weeks did not improve mental well-being (van de Rest et al. 2008). Further, an 18-month supplementation with DHA compared with placebo failed to slow the rate of cognitive and functional decline in a total of 402 patients with mild to moderate Alzheimer's disease (Quinn et al. 2010). These contrasting data do not stand against a supporting role of EPA and DHA for mental health in elderly, whereas they raise questions regarding the therapeutic potential of consuming omega-3 fatty acids to deter the cognitive decline in those suffering from moderate to severe AD (Cederholm and Palmblad 2010).

The cross-sectional nature of our study precludes any assumption about the cause and effect relationship between variables. However, there are data offering mechanisms by which the intake of omega-3 fatty acids potentially benefits functional and structural brain health in late life. For instance, omega-3 fatty acids have anti-inflammatory and vasodilatory properties (Holub and Holub 2004), which may protect general vascular health, and as a secondary effect, maintain blood perfusion of the brain in those whose diets are rich in EPA and DHA. Recently, another mechanism that has gained attention is the finding that DHA, once circulating in the CNS, is available for conversion to neuroprotectin D1 (Bazan et al. 2011). NPD1 elicits neuroprotection in brain (Mukherjee et al. 2004), and, interestingly, has been shown to be deficient in AD brains (Lukiw et al. 2005).

## Limitations

In contrast to the significant association that was observed between the dietary intake of EPA and DHA and the performance on the 7MS (i.e., the cognitive test applied here) and global gray matter, respectively, we did not find such an association when considering plasma proportions of EPA and DHA as an independent variable. One explanation might be that there is likely to be inter-individual fluctuations in circulating concentrations of these fatty acids, which means that there is a potential mismatch between the proportion of these fatty acids in phospholipids and their plasma blood levels. Another limitation is that this study does not detail whether dietary intake of EPA and DHA promotes brain health; it only describes an association between the two characteristics. The reader should also bear in mind that the associations between the dietary intake of EPA and DHA and brain health obtained in the primary study population (i.e., in 252 elderly) included a subsample of subjects who, based on the Goldberg equation (Black 2000), apparently did not report their dietary intake properly. This may potentially have confounded our main results. However, although weaker regarding its significance, the same associations were demonstrated after exclusions of non-adequate reporters (i.e., in 198 elderly). This suggests that the inclusion of this subsample is unlikely to bias our main results. Further, the reader should consider that there was an interval of 5 years between the assessment of dietary intake of EPA and DHA and the 7MS and brain volume measurements. As a result, no definite conclusions can be drawn as to whether the participants followed a similar dietary pattern at the age of 75 years, as they did at the age of 70 years. Finally, confounds by other factors not considered in the present study cannot be excluded.

## Conclusions

Our results provide a potential link between consumption of diets rich in EPA and DHA and enhanced mental health in the elderly. However, this preliminary finding warrants further randomized prospective trials, including repeated measures of cognitive function and brain structure in both cognitively healthy elderly and patients with cognitive impairment. Interestingly, EPA and DHA are mainly found in fish and food of marine origin, which constitutes an important part of the Mediterranean diet (Sjogren et al. 2010). Thus, it cannot be ruled out that the positive association between the dietary intake of EPA and DHA and brain health observed in our elderly study population is driven by the adherence to an healthy lifestyle, rather than by these nutritional factors alone. Further, when evaluating the positive association between omega-3 fatty acid intake and brain measures, it must be born in mind that the beneficial effects are relatively small, as compared to those related to other lifestyle factors, such as education (Le Carret et al. 2003) and physical activity (Weuve et al. 2004). We did not find any association between the dietary intake of EPA and DHA, cognitive function, and brain structure in the cognitively impaired subgroups. Thus, the reader must be careful in making assumptions about the influence of the dietary intake of EPA and DHA on cognitive performance and brain structure in cognitively impaired patients.
